# Reversibility of motor dysfunction in the rat model of NGLY1 deficiency

**DOI:** 10.1186/s13041-021-00806-6

**Published:** 2021-06-13

**Authors:** Makoto Asahina, Reiko Fujinawa, Hiroto Hirayama, Ryuichi Tozawa, Yasushi Kajii, Tadashi Suzuki

**Affiliations:** 1 T-CiRA Discovery, Takeda Pharmaceutical Company Ltd., Fujisawa, Kanagawa 2518555 Japan; 2 Glycometabolic Biochemistry Laboratory, RIKEN Cluster for Pioneering Research, RIKEN, 2-1 Hirosawa, Wako, Saitama 351-0198 Japan; 3 Takeda-CiRA Joint Program (T-CiRA), Fujisawa, Kanagawa 2518555 Japan

**Keywords:** Ngly1, Motor dysfunction, Ngly1 deficient rats

## Abstract

*N-*glycanase 1 (NGLY1) deficiency is a rare inherited disorder characterized by developmental delay, hypolacrima or alacrima, seizure, intellectual disability, motor deficits, and other neurological symptoms. The underlying mechanisms of the NGLY1 phenotype are poorly understood, and no effective therapy is currently available. Similar to human patients, the rat model of NGLY1 deficiency, *Ngly1*−/−, shows developmental delay, movement disorder, somatosensory impairment, scoliosis, and learning disability. Here we show that single intracerebroventricular administration of AAV9 expressing human NGLY1 cDNA (AAV9-hNGLY1) to *Ngly1*−/− rats during the weaning period restored NGLY1 expression in the brain and spinal cord, concomitant with increased enzymatic activity of NGLY1 in the brain. hNGLY1 protein expressed by AAV9 was found predominantly in mature neurons, but not in glial cells, of *Ngly1*−/− rats. Strikingly, intracerebroventricular administration of AAV9-hNGLY1 normalized the motor phenotypes of *Ngly1*−/− rats assessed by the rota-rod test and gait analysis. The reversibility of motor deficits in *Ngly1*−/− rats by central nervous system (CNS)-restricted gene delivery suggests that the CNS is the primary therapeutic target organs for NGLY1 deficiency, and that the *Ngly1*−/− rat model may be useful for evaluating therapeutic treatments in pre-clinical studies.

**Supplementary Information:**

The online version contains supplementary material available at 10.1186/s13041-021-00806-6.

## Introduction

*N-*glycanase 1 (NGLY1), also known as peptide:*N-*glycanase, is an evolutionarily conserved enzyme among eukaryotes that plays a crucial role in quality control for newly synthesized *N-*glycoproteins [1, 2]. *N-*glycanase 1 (NGLY1) deficiency is a rare inherited disorder that is caused by mutations in the *NGLY1* gene. In 2012, the first patient harboring mutations in *NGLY1* was identified [3]. Since then, more than 60 similar patients have been confirmed worldwide and several clinical reports have been published [4–10]. NGLY1 deficient patients show a broad spectrum of clinical features including developmental delay, hypolacrima or alacrima, seizure, intellectual disability, and motor deficits [4–14]. The mutant form of Ngly1 and its orthologs have been analyzed to elucidate the molecular function of NGLY1 in various organisms [1, 15–31]. However, the pathological mechanisms leading to the varied symptoms of NGLY1 deficiency remain obscure, and no effective therapy is currently available.

Our previous study demonstrated that, similar to human patients, *Ngly1−/−* rats show developmental delay, movement disorder, somatosensory impairment, scoliosis, and learning disability [32]. Histological analysis identified prominent pathological abnormalities including necrotic lesions, mineralization, intra- and extra-cellular eosinophilic bodies, astrogliosis, microgliosis, and significant loss of mature neurons in the thalamus of *Ngly1*−/− rats [32]. In endoplasmic reticulum-associated degradation processes, NGLY1 cleaves *N-*glycans from misfolded glycoproteins in the cytosol while they are degraded by the proteasome; the loss of Ngly1 led to accumulation of cytoplasmic ubiquitinated proteins, a marker of misfolded proteins, in the neurons of the central nervous system (CNS) in *Ngly1*−/− rats [32]. In addition, *Ngly1*−/− rats showed axonal degeneration in peripheral nerves [32], similar to human patients.

The face validity of *Ngly1*−/− rats as a model animal for NGLY1 deficiency has been verified by phenotypic analysis [32]. However, to utilize *Ngly1−/− *rats as an animal model for exploring therapeutic options, it is necessary to evaluate the reversibility of symptoms. In addition, it is important to clarify the target organs and cells for the development of therapeutic agents and modalities for NGLY1 deficiency. While Ngly1 is ubiquitously expressed throughout the body with the highest expression in the testis [33], the neurological symptoms in most patients suggest abnormalities in the CNS [4–11, 14]. However, it remains unclear whether loss of NGLY1 in the CNS primarily causes the symptoms of NGLY1 deficiency.

The adeno-associated virus (AAV) is a powerful tool for gene delivery, with features that include the ability to infect different tissues with various injection routes, long-term foreign gene expression, and a lack of pathogenicity in animal models [34]. AAV vectors are also the leading platform for gene delivery for the treatment of a variety of human diseases, with approximately 200 interventional clinical trials involving AAV in human patients registered at ClinicalTrials.gov [35]. In the present study, we investigated whether a single intracerebroventricular (i.c.v.) administration of AAV9 expressing human NGLY1 cDNA would normalize the most apparent and translatable symptoms of *Ngly1*−/− rats. By expressing the hNGLY1 gene specifically in the CNS of *Ngly1*−/− rats by i.c.v. injection, we verified that the motor symptoms are reversible. Our findings clearly demonstrate that the *Ngly1*−/− rat model is useful for evaluating therapeutic options in pre-clinical studies, and suggest the CNS as a target organ for therapies for NGLY1 deficiency.

## Results

### NGLY1 transgene expression pattern after intracerebroventricular injection of viral vectors

**Fig. 1:**
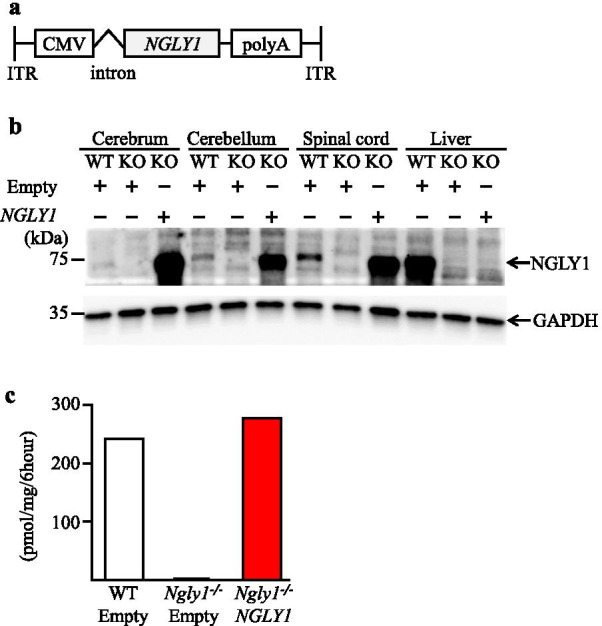
Protein expression and enzymatic activity of hNGLY1 in AAV9-hNGLY1-injected *Ngly1−/*− rats. Protein expression and enzymatic activity of hNGLY1 were restored in the CNS after intracerebroventricular injection of AAV9-hNGLY1 into Ngly1−/− rats. **a** The single-stranded AAV9.hNGLY1 construct. ITR; inverted terminal repeat, CMV; cytomegalovirus enhancer, polyA; human growth hormone polyadenylation signal, *NGLY1*; cDNA of human NGLY1 without 3’UTR. **b** Western blot of endogenous and exogenous NGLY1 protein levels in AAV-injected rats. **c** Enzymatic activity of NGLY1 proteins in the brains of AAV-injected rats (n = 1). WT; wild type rats, KO; *Ngly1−/*− rats. hGH poly(A) signal. Human growth hormone polyadenylation signal

In this study, we aimed to evaluate the reversibility of disease phenotypes in *Ngly1−/− *rats. To this end, a recombinant AAV9 vector expressing cDNA of the human NGLY1 gene under the constitutive CMV promoter (AAV9-hNGLY1) was constructed (Fig. 1a). Although which organs/tissues should be therapeutically targeted remain unclear, given the prominent neurological symptoms [3–10, 14], we evaluated the direct effect of CNS-focused gene delivery to reverse motor dysfunction in *Ngly1−/− *rats.

Our previous analysis of *Ngly1−/− *rats and mice indicated that neuroinflammation, as characterized by increased expression of glial fibrillary acidic protein (GFAP) and ionized calcium-binding adaptor molecule 1 (IbaI) in the thalamus, is one of the earliest phenotype observed [31, 32]. This phenotype is prominent in *Ngly1−/− *rats at 5 weeks of age, but not at 2 weeks of age [32], suggesting that a pathological change occurs between 2 to 5 weeks. We therefore chose 3 weeks of age as the timing for hNGLY1 gene delivery through i.c.v. injection.

Accordingly, purified AAV9-hNGLY1 or empty viral vectors (2 × 10^10^ genome copies/10 μl/rat) were intracerebroventricularly injected into WT and *Ngly1−/− *rats at 3 weeks of age. NGLY1 protein expression was then examined at 8 weeks after AAV9-hNGLY1 or control AAV9 administration. As shown in Fig. 1b, NGLY1 was detected by immunoblotting in the cerebrum, cerebellum, spinal cord, and liver of WT rats, whereas hNGLY1 expression was detected in the cerebrum, cerebellum, and spinal cord of AAV9-hNGLY1-i.c.v. injected *Ngly1−/− *rats. In sharp contrast, hNGLY1 protein was not detected in the liver (Fig. 1b), indicating no detectable leakage of AAV9-hNGLY1 to non-neural organs. The levels of NGLY1 enzymatic activity in the whole brain lysate were also analyzed. The activity assay indicated that AAV9-hNGLY1 administration restored NGLY1 enzymatic activity at comparable levels to that of wild-type rats in the brains of *Ngly1−/− *rats (Fig. 1c). On the other hand, there were the differences in band intensities between endogenous rat NGLY1 and AAV-mediated exogenous human NGLY1 (Fig. 1b). We believe that this is due to the higher reactivity of the antibody to human NGLY1 when compared to that to rat protein, as the antibody was raised against the human NGLY1 peptide.

**Fig. 2:**
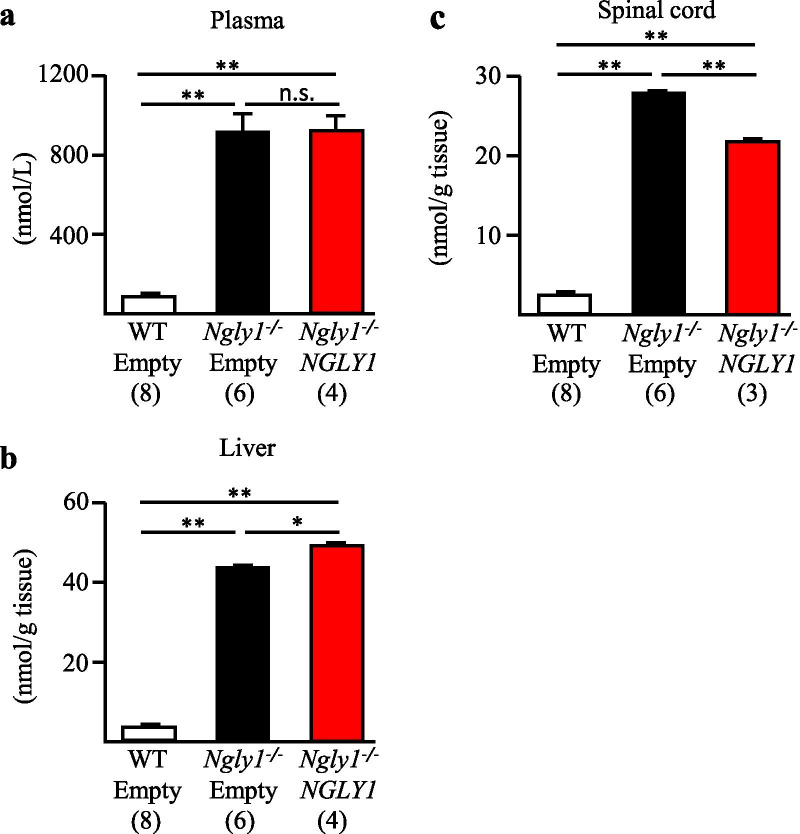
AsnGlcNAc levels in plasma, livers, and spinal cords of AAV9-hNGLY1-injected *Ngly1−/*− rats. AsnGlcNAc levels in plasma (**a**), livers (**b**), and spinal cords (**c**) of 11-week-old virus-injected WT or *Ngly1−/*− rats. Values represent mean ± S.E.M. The number of rats examined is shown in parentheses. *P < *0.05*, **P < *0.01*. n.s. = no significance

Aspartylglycosamine (AsnGlcNAc) was recently identified as a potential biomarker of NGLY1 deficiency in human patients [36]. Consistent with NGLY1-deficient humans, control *Ngly1−/− *rats (injected with empty vector) showed vastly increased AsnGlcNAc levels in the plasma, liver, and spinal cord compared to control WT rats at 11 weeks of age (Fig. 2). Ngly1 catalyzes the cleavage of the amide bond between proximal *N*-acetylglucosamine residues in *N*-glycans and the asparagine residue of a protein [2]. Asn-GlcNAc is predicted to be generated from misfolded glycoproteins by the action of ENGase and proteases in NGLY1−/− cells [37]. It is, therefore, reasonable to assume that AsnGlcNAc level is an indicator of NGLY1 enzymatic activity in vivo. AAV9-hNGLY1-injected *Ngly1−/− *rats showed significantly reduced AsnGlcNAc in the spinal cord (21%), but no such reduction in the plasma or liver, compared to AAV9-empty vector-injected *Ngly1−/− *rats (Fig. 2). A similar result was observed for AsnGlcNAc level in brain tissue in one rat in each group (Additional file 1: Fig. 1). These results suggest that this transgene functions as *N-*glycanase in *Ngly1−/− *rats, leading to reduction of AsnGlcNAc in tissues where the human NGLY1 transgene is expressed.

**Fig. 3:**
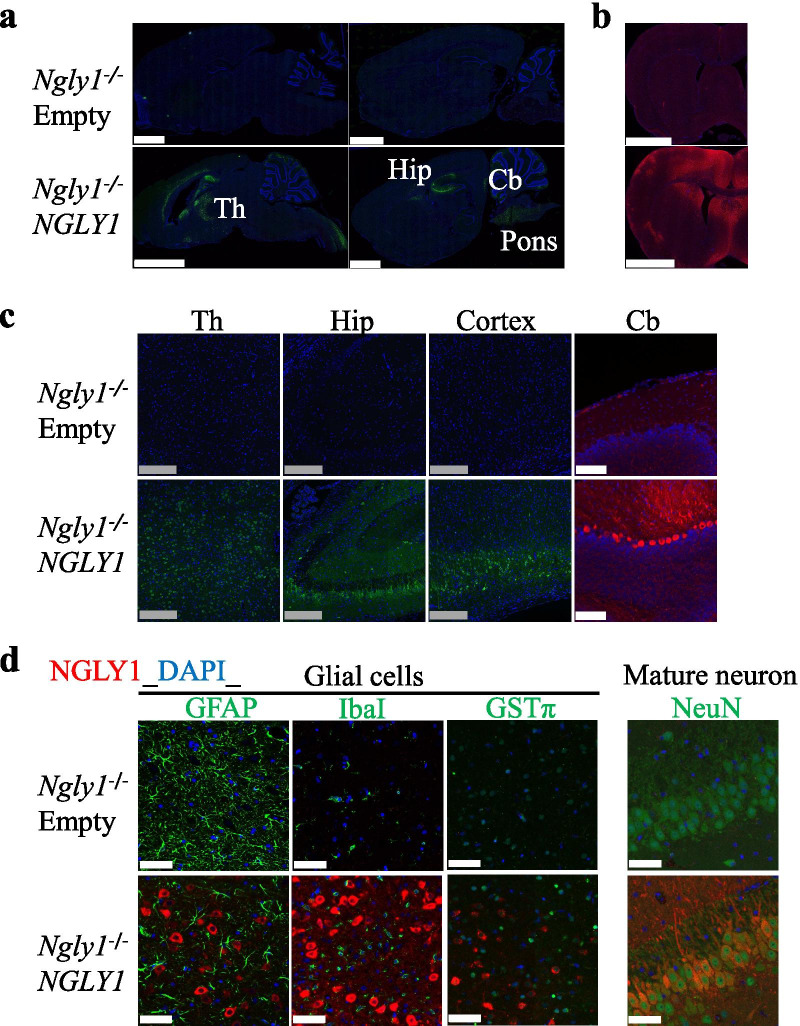
Distribution of hNGLY1 protein in brains after intracerebroventricular injection of AAV9-hNGLY1 in *Ngly1−/*− rats. **a–c** Nuclei were stained with DAPI (blue). Green or red signals indicate hNGLY1-positive cells. **a**, **b** Representative sagittal (**a**) and coronal (**b**) brain sections from 11-week-old AAV-injected *Ngly1−/*− rats. Scale bar = 2.5 mm. **c** Higher magnification images of NGLY1-positive cells across various brain regions after i.c.v. administration. Gray scale bar = 0.25 mm, white scale bar = 0.1 mm. **d** Immunohistochemical staining with hNGLY1 (red) and glial or neuronal markers (green) in the brains of 11-week old AAV-injected rats, using antibodies to Ngly1, GFAP (a marker for astrocytes), IbaI (a marker for microglia), GSTP1 (a marker for oligodendrocytes), and NeuN (a marker for mature neurons). Nuclei have been stained with DAPI (blue). Scale bar = 50 μm

Histological analysis revealed NGLY1 staining throughout the brains of AAV9-hNGLY1 treated *Ngly1−/− *rats (Fig. 3a, b). NGLY1 expression was further confirmed by immunostaining in the pons, thalamus, hippocampus, cerebral cortex, and cerebellar Purkinje cells of AAV9-hNGLY1-injected *Ngly1−/− *rats (Fig. 3a–c). The results were almost equivalent to the distribution pattern of the AAV9-mediated transgene via i.c.v. administration in the brains of adult SD rats [38]. Next, we examined in greater detail what cell types express the transgene after i.c.v. injection of AAV9-hNGLY1. As shown in Fig. 3d, NGLY1 was almost exclusively co-stained with the mature neural cell marker NeuN, but not with glial markers. These results indicate that the transgene introduced into the cerebroventricular space at postnatal week three was preferentially expressed in mature neurons of AAV9-hNGLY1-injected *Ngly1−/− *rats. Notably, this result is inconsistent with a previous study that showed that the transgene was expressed in both mature neurons and glial cells of AAV9-i.c.v. injected adult SD rats [38]. There was no significant difference among the four groups in plasma liver enzymes, AST, or ALT, indicating that liver toxicity was not obvious after i.c.v. injection of AAV9 vectors (Additional file 1: Fig. 2).

### Gene transfer restored motor dysfunction in *Ngly1*−/− rats

**Fig. 4:**
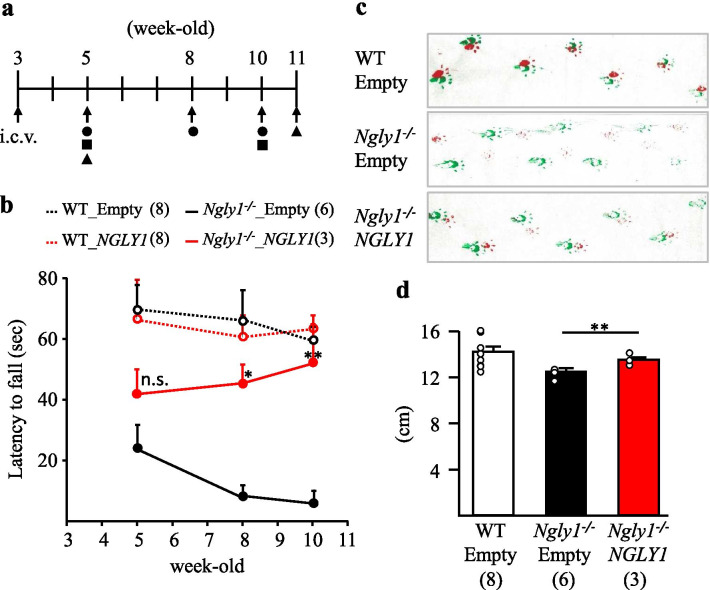
Intracerebroventricular AAV9-hNGLY1 administration attenuated motor deficits in *Ngly1−/*− rats. **a** Experimental paradigm. Rats’ motor function was analyzed for eight weeks after virus injection. Circle; rota-rod test, square; gait analysis, triangle; grip-strength test. **b** Accelerating rota-rod testing for motor coordination of AAV-injected *Ngly1−/−* and WT rats at several ages. The time until falling from the accelerating rod (4–40 rpm for 4 min) is measured as the latency time to fall. **c** Representative paw placement records of 10-week-old AAV-injected rats. **d** Stride lengths of 10-week-old rats in the gait analysis 7 weeks after AAV-injection. Values represent mean ± S.E.M. The number of rats examined is shown in parentheses. *P < *0.05*, **P < *0.01*. n.s. = no significance

Almost all NGLY1-deficient patients show various movement deficits starting in early childhood [3–8, 10, 11]. Our previous study showed that *Ngly1−/− *rats develop motor deficits similar to human patients [32]. To assess the effect of AAV9-hNGLY1 i.c.v. injection on motor function in *Ngly1−/− *rats, various tests (rota-rod test, grip-strength test, and gait analysis) were carried out (Fig. 4a).

#### Rota-rod test

Motor coordination and balance were assessed by the rota-rod test. Strikingly, the latency time to fall was significantly restored in *Ngly1−/− *rats 5 and 7 weeks after AAV9-hNGLY1 viral vector administration relative to the empty viral vector, whereas there was no significant change in latency time 2 weeks after administration (Fig. 4b). Notably, 7 weeks after administration, the motor performance of AAV9-hNGLY1-injected *Ngly1−/− *rats on the rota-rod tests was similar to that of age-matched viral-injected WT rats (Fig. 4b).

#### Gait analysis

*Ngly1*−/− rats exhibit obvious gait abnormalities, including a wide-based ataxic gait, shorter stride lengths, and increased stance ratio, compared with WT rats [32]. In this study, AAV9-hNGLY1 viral vector administration apparently ameliorated gait abnormalities in *Ngly1−/− *rats at 7 weeks after administration (Fig. 4c), concomitant with restoration of stride length, when compared with empty viral vector-injected *Ngly1−/− *rats (Fig. 4d). There was, however, no significant difference in stride length between AAV9-hNGLY1-injected and empty vector-injected *Ngly1*−/− rats at 2 weeks after administration (Additional file 1: Fig. 3).

#### Grip-strength test

The grip strength of the forelimbs (two paws) or forelimbs and hindlimbs (four paws) are significantly reduced in *Ngly1*−/− rats compared with WT rats [32]. In this study, the grip strengths of two paws or four paws were not significantly recovered in AAV9-hNGLY1 vector-injected *Ngly1*−/− rats compared with empty vector-injected *Ngly1*−/− rats even at 8 weeks after administration (Additional file 1: Fig. 4). There was no significant difference in body weight between AAV9-hNGLY1-injected rats and control AAV9 vector-injected rats in either the WT or *Ngly1−/−*genotype group (Additional file 1: Fig. 5).

### Gene transfer partially ameliorates neuroinflammation in *Ngly1*−/− rats

**Fig. 5:**
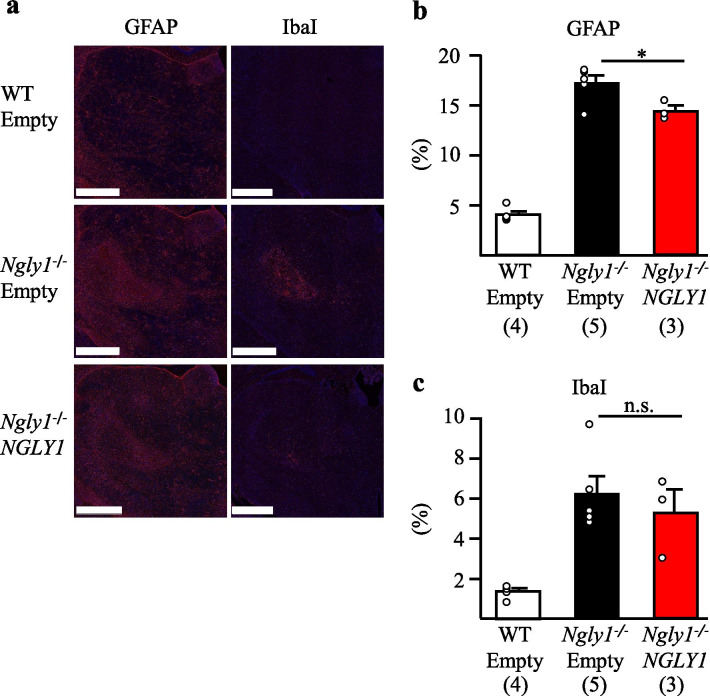
Intracerebroventricular AAV9-hNGLY1 administration partly attenuated astrogliosis in thalamic VPL/VPM regions of *Ngly1−/*− rats. **a** Immunohistochemistry for GFAP and IbaI in the thalamus of virus-injected WT and *Ngly1−/*− rats at 11 weeks of age. Scale bar = 0.1 mm. Nuclei were stained with DAPI. **b, c** Quantitative analyses show the area occupied by GFAP (**b**) or IbaI (**c**) positive areas in the thalamic VPL/VPM regions of virus-injected WT and *Ngly1−/*− rats. Values represent mean ± S.E.M. The number of rats examined is shown in parentheses. *P < *0.05*. n.s. = no significance

Neuroinflammation, which is characterized by increased expression of GFAP and IbaI, is commonly observed in the thalamus of *Ngly1−/− *rodents, and is particularly prominent in the thalamic nuclei at the VPM/VPL regions [31, 32]. Because reactive astrocytes and microglial activation are often regarded as indications of neural toxicity or neuronal death in neurodegenerative animal models [39], we attempted to quantify GFAP and IbaI by immunofluorescence staining at 11 weeks of age. Compared with empty vector-injected *Ngly1−/*− rats, AAV9-hNGLY1-injected *Ngly1−/*− rats showed significantly reduced GFAP level in the VPM/VPL regions of the thalamus (Fig. 5a, b). However, there was no notable change in microglial activation, as indicated by enhanced immunoreactivity of IbaI, in regions of the thalamus of AAV9-hNGLY1-injected *Ngly1−/*− rats (Fig. 5a, c). *Ngly1−/*− rats showed characteristic neurodegenerative pathological abnormalities in the H&E-stained thalamic sections [32]. There was no recognizable alleviation of these characteristics in hematoxylin and eosin (H&E) stained sections of AAV9-hNGLY1-injected *Ngly1−/*− rats (Additional file 1: Fig. 6). Overall, despite observing clear reversibility of motor dysfunction in AAV9-hNGLY1-injected *Ngly1−/*− rats, some neuroinflammation phenotypes were not corrected by treatment, as far as we were able to observe. At present, how neuroinflammation contributes to abnormal motor function in *Ngly1−/*− rats remains unclear.

## Discussion

To explore therapeutic options for intractable diseases where the pathophysiological cascade from causal gene to symptoms remain unclear, it is important to establish animal models that show reversibility of symptoms in order to identify the therapeutic target organs and therapeutic windows of treatment. In the present study, we revealed that a single intracerebroventricular administration of AAV9 expressing human NGLY1 cDNA into *Ngly1*−/− rats restored NGLY1 expression in the CNS, increased the enzymatic activity of NGLY1, and normalized motor function. These results suggest that *Ngly1*−/− rats are useful for evaluating therapeutic agents and modalities for NGLY1 deficiency in pre-clinical tests. Our study also clearly demonstrated that the CNS is associated with motor dysfunction in this rat model, suggesting the CNS as a possible therapeutic target organ for NGLY1 deficiency—at least for motor function-related symptoms. At the cellular level, NGLY1 deficiency seems to lead to increased stress responses related to quality control of glycoproteins; however, it is still unclear how such stress responses result in functional deficiency that causes the symptoms. Our observations suggest that specific cell types in the CNS are functionally vulnerable to such stress responses and cause motor dysfunction in NGLY1 deficiency patients, which could be improved by gene therapy at the appropriate timing after birth.

Our analysis showed that motor performance in the rota-rod test and stride length in gait analysis drastically improved with age in *Ngly1*−/− rats that received AAV9-hNGLY1 relative to the empty vector. Although NGLY1 enzymatic activity in the brain of AAV9-hNGLY1-injected *Ngly1−/*− rats was comparable to that of WT rats 8 weeks after administration, the ataxic gait pattern and limb grip strengths in *Ngly1−/*− rats did not improve. In general, it takes approximately 10–14 days for an AAV-mediated transgene to reach the highest level of gene expression, depending on the capsid serotype and the tissue of delivery. The efficiency of transgene expression in the early period after administration may have been too low to recover the ataxic gait pattern and grip strength of limbs in *Ngly1−/*− rats. To examine how further increases in the NGLY1 gene expression level affect motor performance in *Ngly1−/*− rats, it will be necessary to optimize the experimental conditions, such as verifying the effect of codon optimization, the introduction of a self-complementary vector [40], and inserting terminal sequences, such as woodchuck hepatitis virus posttranscriptional regulatory element [41], for the rapid induction of mRNA and protein expression of transgenes. Optimization of additional factors, such as the timing of injections, dose of viruses, motor learning, or the observation period after AAV9-hNGLY1 administration, will be required to achieve further recovery of motor functions.

In this study, the transduction profile of hNGLY1 was almost exclusively in mature neural cells, but not in glial cells. This result suggested that the induction of hNGLY1 into neural cells would rescue motor dysfunction in *Ngly1−/*− rats. Donsante et al. reported that the transduction profile is in both mature neurons and astrocytes, when the AAV9-mediated GFP was introduced under the mini-chicken β-actin promoter via i.c.v. administration in adult SD rats [38]. On the other hand, Shervin et al. reported that the transduction profile is a primarily neuronal and astrocytes are poorly transduced, when the AAV9-mediated GFP was introduced under the CMV promoter via i.c.v. administration in 3-week old mice [42]. Thus, we think that the transduction profile may depend on age at the time of i.c.v. administration or the promoter used.

By expressing the *hNGLY1* gene specifically in the CNS of *Ngly1−/*− rats by i.c.v. injection, we identified the CNS—including the brain and spinal cord—as possible therapeutic target organs for NGLY1 deficiency, which is critical information for developing therapeutic options. It is unclear what part of the CNS is most closely associated with the symptoms of Ngly1 deficiency. Furthermore, although neuroinflammation is one of the earliest defects observed in the thalamus of rodent models of NGLY1 deficiency, it is unclear whether neuroinflammation is associated with motor dysfunction in *Ngly1−/*− rodents [31, 32]. Compared with AAV9-empty vector-injected *Ngly1−/*− rats, GFAP expression levels were significantly ameliorated in the thalamus of AAV9-hNGLY1-injected *Ngly1−/*− rats at 11 weeks of age when their motor function was recovered. However, IbaI expression levels in the thalamus were not altered. Furthermore, there was no alleviation of neurodegenerative pathological abnormalities observed in the H&E-stained thalamic sections of AAV9-hNGLY1-injected *Ngly1−/*− rats. Thus, the thalamus may not be associated with motor dysfunction in *Ngly1−/*− rats.

In the previous report, *Ngly1−/*− rats showed accumulation of ubiquitinated proteins in the brain and spinal cords [32]. However, there was no obvious difference in ubiquitin-positive signals of the brain between the AAV9-NGLY1-injected and empty vector-injected *Ngly1*−/− rats (Additional file 1: Fig. 7a). In the spinal cord, there was no significant difference in the poly-ubiquitinated proteins between the AAV9-NGLY1-injected and empty vector-injected *Ngly1*−/− rats (Additional file 1: Fig. 7b, c). Previous study revealed that *Ngly1*-KO rats exhibited axonal loss in sciatic nerve at 29 weeks of age, but not at 5 weeks of age [32]. The axonal loss, on the other hand, did not affect myelination in sciatic nerves, which could relate to motor function, even at 29 weeks of age [32]. In this study, we evaluated motor function up to 11 weeks of age and did not examine pathologies in the sciatic nerve. The hNGLY1 protein were introduced to the cerebellum of AAV9-hNGLY1-injected *Ngly1*−/− rats. Previous study reported that *Ngly1*−/− rats did not show any pathological abnormalities including accumulation of ubiquitinated proteins, astrogliosis, and microgliosis in the cerebellum which affects motor function [32]. We therefore did not examine the cerebellum in this study. The introduced hNGLY1 protein in the brain of AAV9-hNGLY1-injected *Ngly1−/*− rats was primarily observed in cerebellar Purkinje cells, hippocampus, cerebral cortex, pons, and brain regions other than the thalamus. Further studies will be required to identify the brain region responsible for impaired motor function in NGLY1 deficiency model rodents.

This study has presented a potential therapeutic intervention for NGLY1 deficiency in a rodent model. We demonstrated the reversibility of motor deficits in Ngly1−/− rats after CNS-restricted gene delivery, suggesting that this rat model is useful for evaluating treatments in pre-clinical studies. Our study also identified the CNS as a potential therapeutic target organ for NGLY1 deficiency, and i.c.v. administration as a promising administration route for treatment of this genetic disorder. The next step will be to optimize the dose/timing of gene transfer, as well as to determine the therapeutic window for effective therapy. To apply its clinical significance brought by motor function recovery to other symptoms, the further study will be needed to verify whether AAV9-mediated hNGLY1 gene delivery would improve the learning ability which is evaluated by water maze tests. Further clarification of the underlying disease pathophysiology will also allow us to develop more effective and precise therapeutic approaches for treating Ngly1 deficiency.

## Methods

### Animals

All animal care procedures and experiments conformed to the Association for Assessment and Accreditation of Laboratory Animal Care guidelines and were approved by the Experimental Animal Care and Use Committee of Takeda Pharmaceutical Company Limited and Axcelead Drug Discovery Partners, Inc. All rats were housed in individual cages in a room with controlled temperature (23 °C), humidity (55%), and lighting (lights on from 7:00 am to 7:00 pm) and were fed a normal chow diet (CLEA Japan; CE2 diet) with free access to water. Rats were sacrificed by exsanguination. The organs were then removed and weighed.

### Viral production and injections

cDNA sequence of NGLY1 was amplified by PCR using pCMV6-huPNGase-WT (our laboratory stock) as a template and primers (forward primer 5′-AATTCCCCGGGGATCCATGGCGGCGGCGGCATTG, reverse primer 5′-GCTTCTGCAGGTCGACTCAAAGGTCACTGAATTTTATA). The resultant NGLY1 fragment was cloned into pAAV-MCS (Cell Biolabs, Inc.; VPK-410) digested with BamHI and SalI using In-Fusion HD cloning kit (TaKaRa Bio; 639648) according to the manufacturer’s protocol. AAV9 was used under a non-exclusive research license agreement with REGENXBIO Inc. The pAAV-hNGLY1 vectors, pHelper vectors (Cell Biolabs, Inc.; 340202), and pAAV2/9n vectors were transfected into 293 cells (Takara Bio; #632273) by Xfect transfection reagent (Takara Bio; 63118) and purified by commercial kits according to the manufacturer’s recommended protocol (Takara Bio; #6666). Vector preparations were titered by quantitative PCR (Takara Bio; #6233). A recombinant AAV9 vector expressing cDNA of the human NGLY1 gene was under the constitutive promoter from cytomegalovirus. AAV9-hNGLY1- or AAV9-empty viral vectors were unilaterally intracerebroventricularly injected at a dose of 2 × 10^10^ genome copies (GC)/rat in a final volume of 10 μl *Ngly1−/*− rats and littermate WT rats at 3 weeks of age under 2% isoflurane anesthesia (the dose/kg in each group; WT rats + empty: 42 × 10^10^ ± 2.3 GC/kg, WT + hNGLY1: 42 × 10^10^ ± 2.4 GC/kg, *Ngly1−/*− rats + empty: 37 × 10^11^ ± 5.7 GC/kg, *Ngly1−/*− rats + hNGLY1: 38 × 10^11^ ± 0.72 GC/kg into *Ngly1−/*− rats). The coordinates for i.c.v. injection were adjusted by the length between interaural line and bregma, following the text [43]. After injection, rats were weaned and returned to their cages.

### Motor function tests

#### Rota-rod test

Motor performance and coordination were tested using an accelerating rota-rod (Muromachi Kikai Co., Ltd.; MK610) at different ages. Each rat underwent 2 days of training sessions and a 1-day test session. On the training days, the rats completed four separate trials of 1 min each, walking at 4 rpm with a 20 min interval between trials. On day 3, the test was performed comprising four separate trials separated by 20 min intervals. During the test session, the speed of the rota-rod was accelerated from 4 to 40 rpm over the course of 4 min, and the time taken for the rats to fall from the rod was measured. If the rats stayed on the rod until the end of the 4 min trial, a time of 240 s was recorded.

#### Gait analysis

To assess changes in gait, gait analysis was carried out. The rats’ forepaws and hind paws were coated with two non-toxic water-soluble inks (forepaws in green, hind paws in red). The rats were then allowed to walk along a 100 cm long, 15 cm wide runway. The floor of the runway was covered with sheets of white paper. The footprints were manually analyzed, and the stride length of each paw and the stance ratio (width ratio; hind to forelimbs) were quantified.

#### Grip-strength test

For the grip-strength test, each rat was lifted by its tail and its forelimbs (or all limbs) were allowed to grasp metal mesh fixed to a force-electricity transducer (Brain Science Idea Co. Ltd.; BS-TM-RM). The rat was gently pulled upward while it grasped the mesh with its forelimbs (or all limbs). The maximal force reached immediately before the rat released the mesh was taken as the grip strength.

### Histological analysis

For light microscopy analysis, tissues from rats at 11 weeks of age were dissected and fixed in 4% paraformaldehyde.

Some brains were trimmed coronally based on STP position paper at level 2 [44], paraffin-embedded, sectioned at 4–6 μm thickness, and mounted onto slides. Sections were stained with H&E or used for immunohistochemical analysis. Other brains were trimmed sagittally in two patterns, sectioned at 10 μm thickness, and mounted onto slides as frozen sections for immunohistochemical analysis.

### Preparation of cytosolic fraction and enrichment of NGLY1

For preparation of lysate, 50 mg of rat brain was sliced and resuspended in 500 µl NGLY1 buffer (100 mM Tris–HCl; pH 7.5, 250 mM sucrose, 1 mM Pefabloc™SC (Sigma-Aldrich; 11429868001), and 1 × cOmplete™ protease inhibitor cocktail (Sigma-Aldrich; 11836145001)). Tissue was homogenized using a Biomasher-II (Nippi-inc; 320 103) by 4 × 20 s homogenization with intervals of 20 s on ice. The sample was centrifuged at 20,000×*g* for 5 min at 4 °C and the supernatant was transferred to a new tube. The sample was further separated by ultracentrifugation (100,000×*g* for 30 min at 4 °C) and the supernatant was collected as a cytosolic fraction. Enrichment of NGLY1 from the cytosolic fraction was performed using a Butyl-Sepharose column (GE Healthcare Life Sciences; 17-0980-01) [45]. Briefly, saturated ammonium sulfate solution was added to the cytosolic fraction until the ammonium sulfate concentration reached 220 mM. The suspension was then centrifuged at 20,000×*g* for 10 min at 4 °C and the supernatant was collected. Next, the supernatant was applied to a Butyl-Sepharose column conditioned with equilibration buffer (NGLY1 buffer containing 220 mM ammonium sulfate). The column was washed with equilibration buffer and washing buffer (NGLY1 buffer containing 175 mM ammonium sulfate). Finally, NGLY1 was eluted with 5 ml NGLY1 buffer. The eluted fraction was concentrated using an Amicon Ultra-15 (30,000 molecular weight cut-off; Millipore; UFC903008). The protein concentration was measured using a BCA assay kit (Thermo Scientific; 23227) according to the manufacturer's instructions.

### Activity assay of the enriched NGLY1 fraction

The activity assay of NGLY1 was carried out as previously described [28]. Briefly, the reaction mixture containing 25 µl of the NGLY1 fraction in a total volume of 30 µl containing 1 mM DTT, 1 mM Pefabloc™ SC, and 1 × cOmplete™ protease inhibitor cocktail (EDTA-free) together with 53 pmol of BODIPY-asialoglycopeptide (BODIPY-ASGP) was incubated at 25 °C for 6 h. The reaction was terminated by adding 100 µl of 100% EtOH and centrifugation at 20,000×*g* for 2 min. The resulting supernatant was collected and evaporated to dryness in a Speed-Vac concentrator. The substrate and reaction product were separated by HPLC using an InertSustain C18 HP (3 µm, 3.0 × 150 mm, GL Science; 5020-07425). The elution conditions were as follows: eluent A, DW containing 0.1% Trifluoroacetic Acid (TFA); eluent B, 100% acetonitrile containing 0.1% TFA. The column was equilibrated with eluent A/eluent B (60/40) at a flow rate of 0.45 ml/min. After injecting a sample, the concentration of eluent B was increased linearly from 40 to 95% over 25 min. BODIPY-ASGP was detected by measuring fluorescence (λ excitation 503 nm, λ emission 512 nm).

### Immunoblotting analysis

Brains, spinal cords, and livers were isolated, desheathed in saline, and immediately frozen on dry ice. Lysates were prepared by homogenizing the tissues in T-PER buffer (Thermo Fisher; 78510) with a protease inhibitor cocktail. The protein amounts in the lysates were quantified using a BCA assay kit ((Thermo Scientific; 23227). Tissue lysates (2–10 μg) were denatured at 65 °C and resolved by SDS-PAGE (Bio-Rad; #4561033) and then transferred onto polyvinylidene fluoride membranes (Bio-Rad; #1704157) according to the manufacturer's instructions. Membranes were incubated with the primary antibody followed by incubation with the horseradish peroxidase (HRP)-conjugated secondary antibody. The results were visualized using a ChemiDoc Imaging System with a western chemiluminescent HRP substrate (Merck Millipore; WBKLS0100).

### Immunostaining analysis

For immunohistochemical analysis, all tissue sections were subjected to antigen retrieval using the microwave method (in Tris–EDTA buffer (pH 9) for 15–20 min). After blocking, sections were incubated with primary antibodies overnight at 4 °C, followed by 1 h incubation with fluorescently-labeled secondary antibodies.

### Antibodies

The following antibodies were used for the indicated dilutions. For immunoblotting: rabbit anti-NGLY1 (1:100; ATLAS ANTIBODIES; HPA036825), mouse anti-GAPDH (6C5; 1:5000; Millipore; MAB374), and HRP-conjugated antibody (1:10,000; CST; 7076S). For immunostaining: rabbit anti-GFAP (1:2000; Abcam; ab7260), goat anti-IbaI (1:1000; Abcam; ab5076), rabbit anti-GSTP1 (1:300; GenTex; GTX112695), rabbit anti-NeuN (1:3000; Abcam; ab177487), and mouse anti-NeuN (1:1000; BioLegend; SIG-39860).

### Measurement of plasma ASL and ALT levels

Plasma AST and ALT were measured using commercial kits according to the manufacturer’s instructions (AST_Biovision; #K753-100, ALT_Biovision;K752-100).

### Measurement of plasma and organ AsnGlcNAc levels

AsnGlcNAc levels in plasma and organs were determined by liquid chromatography/tandem mass spectrometry. Detailed protocols were previously described [31].

### Statistical analysis

Data are presented as mean ± SEM. Student’s *t*-test was used to compare variables between AAV9-empty vector-injected *Ngly1−/*− rats and AAV9-hNGLY1-injected *Ngly1−/*− rats. One-way analysis of variance with the Tukey–Kramer multiple comparison test was used for comparisons between three or four groups. Statistical significance is reported as *P < 0.05 and **P < 0.01.

## Supplementary Information

**Additional file 1: Table.** Raw data of rota-rod tests in each animal at each age. **Figure 1.** AsnGlcNAc levels in the brain of 11-week-old viral-injected WT or *Ngly1−/*− rats. Values represent means. One rat is examined in each group.  **Figure 2.** Plasma AST (**a**) and ALT (**b**) levels of 11-week-old virally injected WT or *Ngly1*−*/*− rats. Values represent means ± S.E.M. The number of rats examined is shown in parentheses. n.s. means no significance. **Figure 3.** Stride lengths of 5-week old rats in gait analysis 2 weeks after AAV-injection. Values represent means ± S.E.M. The number of rats examined is shown in parentheses. n.s. means no significance. **Figure 4.** Grip-strength tests for assessment of 2paws (**a**) or 4paws (**b**) muscle force of 11-week-old AAV-injected rats. Values represent means ± S.E.M. The number of rats examined is shown in parentheses. n.s. means no significance. **Figure 5.** Body weight of WT and *Ngly1*−*/*− rats. Rats were weighed weekly after AAV administration. The number of rats examined is shown in parentheses. n.s. means no significance. **Figure 6.** Neuronal degeneration in the thalamus of *Ngly1−*/− rats was not recovered in AAV9-hNGLY1 injected *Ngly1*−/− rats. H&E-stained sections of the thalamus from the WT and Ngly1/rats 8 weeks after AAV administration. Scale bar 250 μm. **Figure 7.** (**a**) Immunohistochemistry for ubiquitin in the thalamus of virus-injected WT and *Ngly1*−/− rats at 11 weeks of age. Scale bar, 250 μm. Nuclei were stained with DAPI. (**b**) Accumulation of polyubiquitinated proteins in spinal cords of *Ngly1*−/− and WT rats. The total protein extracts were separated from the spinal cords of the rats and analyzed by immunoblotting using anti-polyubiquitinated antibodies (top) and anti-GAPDH antibodies (bottom; loading control). (**c**) Semi-quantitative analyses by densitometry were carried out. Values represent mean ± SEM (*Ngly1*−/− rats + empty; n = 5, *Ngly1*−/− rats + hNGLY1; n = 3). n.s. = no significance.

## Data Availability

The datasets generated during and/or analyzed during the current study are available from the corresponding author on reasonable request.
