# Correction to: Reversibility of motor dysfunction in the rat model of NGLY1 deficiency

**DOI:** 10.1186/s13041-021-00825-3

**Published:** 2021-08-16

**Authors:** Makoto Asahina, Reiko Fujinawa, Hiroto Hirayama, Ryuichi Tozawa, Yasushi Kajii, Tadashi Suzuki

**Affiliations:** 1 T-CiRA Discovery, Takeda Pharmaceutical Company Ltd., Fujisawa, Kanagawa 2518555 Japan; 2 Glycometabolic Biochemistry Laboratory, RIKEN Cluster for Pioneering Research, RIKEN, 2-1 Hirosawa, Wako, Saitama 351-0198 Japan; 3 Takeda-CiRA Joint Program (T-CiRA), Fujisawa, Kanagawa 2518555 Japan

## Correction to: Mol Brain (2021) 14:91 https://doi.org/10.1186/s13041-021-00806-6

Following publication of the original article [1], the authors would like to correct 2 errors in the "Methods" section.

The first sentence in the "Preparation of cytosolic fraction and enrichment of NGLY1" subsection originally read:

- For preparation of lysate, 50 mg of rat brain was sliced and resuspended in 500 µl NGLY1 buffer (5 mM Tris–HCl; pH 7.5), 250 mM glycerol, 1 mM Pefabloc™SC (Sigma-Aldrich; 11429868001), and 1 × cOmplete™ protease inhibitor cocktail (Sigma-Aldrich; 11836145001).

The sentence should read (the change has been highlighted in **bold typeface**):

- For preparation of lysate, 50 mg of rat brain was sliced and resuspended in 500 µl NGLY1 buffer (**100** mM Tris–HCl; pH 7.5, 250 mM **sucrose**, 1 mM Pefabloc™SC (Sigma-Aldrich; 11429868001), and 1 × cOmplete™ protease inhibitor cocktail (Sigma-Aldrich; 11836145001)**)**.

The second sentence in the "Activity assay of the enriched NGLY1 fraction" section originally read:

- Briefly, the reaction mixture containing 25 μl of the NGLY1 fraction in a total volume of 30 μl of 10 mM Tris–HCl (pH 7.5), 50 mM sucrose, 1 mM DTT, 1 mM Pefabloc™ SC, and 1 × cOmplete™ protease inhibitor cocktail (EDTA-free) together with 53 pmol of BODIPY- asialoglycopeptide (BODIPY-ASGP) was incubated at 25 °C for 6 h.

The second sentence should read (the change has been highlighted in **bold typeface**):

- Briefly, the reaction mixture containing 25 µl of the NGLY1 fraction in a total volume of 30 µl **containing** 1 mM DTT, 1 mM Pefabloc™ SC, and 1× cOmplete™ protease inhibitor cocktail (EDTA-free) together with 53 pmol of BODIPY-asialoglycopeptide (BODIPY-ASGP) was incubated at 25 °C for 6 h.

These errors do not affect any of the conclusions presented in the article. The original article [1] has been corrected.
