# Supplementary material for: Reversibility of motor dysfunction in the rat model of NGLY1 deficiency
Source: Mol Brain. 2021 Jun 13;14:91. doi: 10.1186/s13041-021-00806-6 (PMC8201687; doi:10.1186/s13041-021-00806-6)
Supplement: Supplementary file 1 — Additional file 1: Table. Raw data of rota-rod tests in each animal at each age. Figure 1. AsnGlcNAc levels in the brain of 11-week-old viral-injected WT or Ngly1−/− rats. Values represent means. One rat is examined in each group. Figure 2. Plasma AST (a) and ALT (b) levels of 11-week-old virally injected WT or Ngly1−/− rats. Values represent means ± S.E.M. The number of rats examined is shown in parentheses. n.s. means no significance. Figure 3. Stride lengths of 5-week old rats in gait analysis 2 weeks after AAV-injection. Values represent means ± S.E.M. The number of rats examined is shown in parentheses. n.s. means no significance. Figure 4. Grip-strength tests for assessment of 2paws (a) or 4paws (b) muscle force of 11-week-old AAV-injected rats. Values represent means ± S.E.M. The number of rats examined is shown in parentheses. n.s. means no significance. Figure 5. Body weight of WT and Ngly1−/− rats. Rats were weighed weekly after AAV administration. The number of rats examined is shown in parentheses. n.s. means no significance. Figure 6. Neuronal degeneration in the thalamus of Ngly1−/− rats was not recovered in AAV9-hNGLY1 injected Ngly1−/− rats. H&E-stained sections of the thalamus from the WT and Ngly1/rats 8 weeks after AAV administration. Scale bar 250 μm. Figure 7. (a) Immunohistochemistry for ubiquitin in the thalamus of virus-injected WT and Ngly1−/− rats at 11 weeks of age. Scale bar, 250 μm. Nuclei were stained with DAPI. (b) Accumulation of polyubiquitinated proteins in spinal cords of Ngly1−/− and WT rats. The total protein extracts were separated from the spinal cords of the rats and analyzed by immunoblotting using anti-polyubiquitinated antibodies (top) and anti-GAPDH antibodies (bottom; loading control). (c) Semi-quantitative analyses by densitometry were carried out. Values represent mean ± SEM (Ngly1−/− rats + empty; n = 5, Ngly1−/− rats + hNGLY1; n = 3). n.s. = no significance. [file 13041_2021_806_MOESM1_ESM.pdf]

Additional file 1

Reversibility of motor dysfunction in the rat model for Ngly1 deficiency.

Makoto Asahina, Reiko Fujinawa, Hiroto Hirayama, Ryuichi Tozawa, Yasushi Kajii, and Tadashi Suzuki

Table

Fig. 1-6

Additional table

Raw data of rota-rod tests in each animal at each age.

5w
Male

Genotype			No.	Trial 1	2	3	4	Average
WT	empty	1	BL10	56	65	64	69	64
		2	R5	20	59	82	89	63
		3	BL8	37	57	59	60	53
		4	Blu1	112	96	83	37	82
		5	R6	43	51	31	48	43
		6	R7	67	44	39	62	53
		7	Blu7	98	112	131	95	109
		8	BL7	96	90	89	90	91
	Ngly1	1	R3	90	105	27	107	82
		2	Blu4	76	98	127	79	95
		3	BL3	49	39	31	26	36
		4	BL5	108	115	152	100	119
		5	R8	61	28	22	34	36
		6	R4	76	93	94	71	84
		7	Blu3	45	64	98	84	73
		8	BL9	11	7	9	2	7
KO	empty	1	Blu4	0	0	0	0	0
		2	Blu8	11	17	20	14	16
		3	12	22	22	28	35	27
		4	13	22	23	25	13	21
		5	14	21	14	38	26	25
		6	16	53	45	60	65	56
	Ngly1	1	R1	61	45	42	47	49
		2	9	43	59	56	47	51
		3	11	17	23	20	41	25

8w
Male

Genotype			No.	Trial 1	2	3	4	Average
WT	empty	1	BL10	63	63	79	83	72
		2	R5	64	48	72	69	63
		3	BL8	20	41	22	40	31
		4	Blu1	77	78	98	92	86
		5	R6	83	108	133	70	99
		6	R7	40	34	38	27	35
		7	Blu7	36	56	43	37	43
		8	BL7	79	104	128	94	101
	Ngly1	1	R3	89	61	86	86	81
		2	Blu4	29	22	76	102	57
		3	BL3	30	28	41	55	39
		4	BL5	63	33	95	38	57
		5	R8	60	40	50	56	52
		6	R4	51	81	95	95	81
		7	Blu3	69	104	76	97	87
		8	BL9	35	29	52	19	34
KO	empty	1	Blu4	0	0	0	0	0
		2	Blu8	9	13	14	12	12
		3	12	0	0	0	0	0
		4	13	16	11	8	17	13
		5	14	0	0	0	0	0
		6	16	32	20	21	21	24
	Ngly1	1	R1	55	64	61	52	58
		2	9	46	31	40	37	39
		3	11	37	43	34	41	39

10w
Male

Genotype			No.	Trial 1	2	3	4	Average
WT	empty	1	BL10	55	43	41	67	52
		2	R5	21	52	78	75	57
		3	BL8	23	54	33	70	45
		4	Blu1	63	32	109	94	75
		5	R6	109	51	83	80	81
		6	R7	37	41	69	65	53
		7	Blu7	26	47	83	78	59
		8	BL7	22	31	84	94	58
	Ngly1	1	R3	45	83	89	109	82
		2	Blu4	80	72	71	91	79
		3	BL3	30	44	52	84	53
		4	BL5	43	83	95	60	70
		5	R8	67	57	83	51	65
		6	R4	38	41	60	88	57
		7	Blu3	42	25	75	87	57
		8	BL9	31	28	50	75	46
KO	empty	1	Blu4	0	0	0	0	0
		2	Blu8	18	10	12	15	14
		3	12	0	0	0	0	0
		4	13	0	0	0	0	0
		5	14	0	0	0	0	0
		6	16	28	18	23	17	22
	Ngly1	1	R1	68	72	98	73	78
		2	9	44	31	45	31	38
		3	11	36	46	42	40	41

Additional file Fig. 1

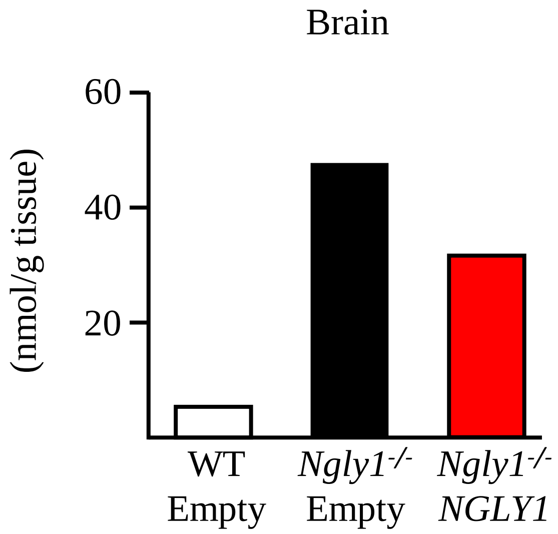

Additional file Fig. 1

AsnGlcNAc levels in the brain of 11-week-old viral-injected WT or Ngly1−/− rats. Values represent means. One rat is examined in each group.

Additional file Fig. 2

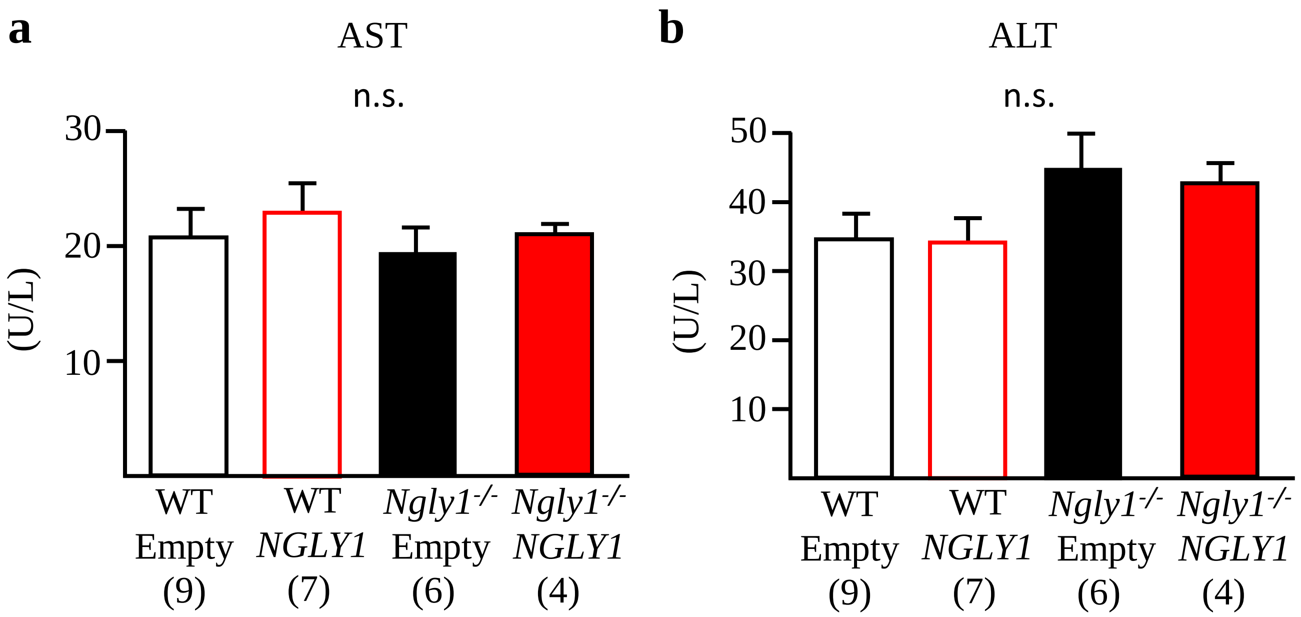

Additional file Fig. 2

Plasma AST (**a**) and ALT (**b**) levels of 11-week-old virally injected WT or Ngly1−/− rats. Values represent means ± S.E.M. The number of rats examined is shown in parentheses. n.s. means no significance.

Additional file Fig. 3

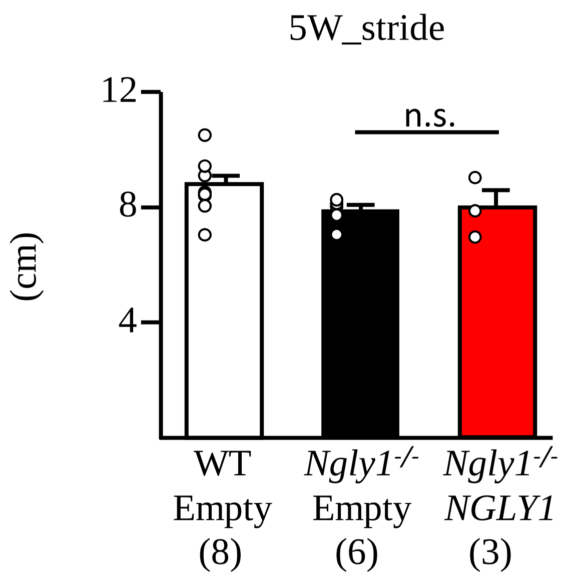

Additional file Fig. 3

Stride lengths of 5-week old rats in gait analysis 2 weeks after AAV-injection. Values represent means ± S.E.M. The number of rats examined is shown in parentheses. n.s. means no significance.

Additional file Fig. 4

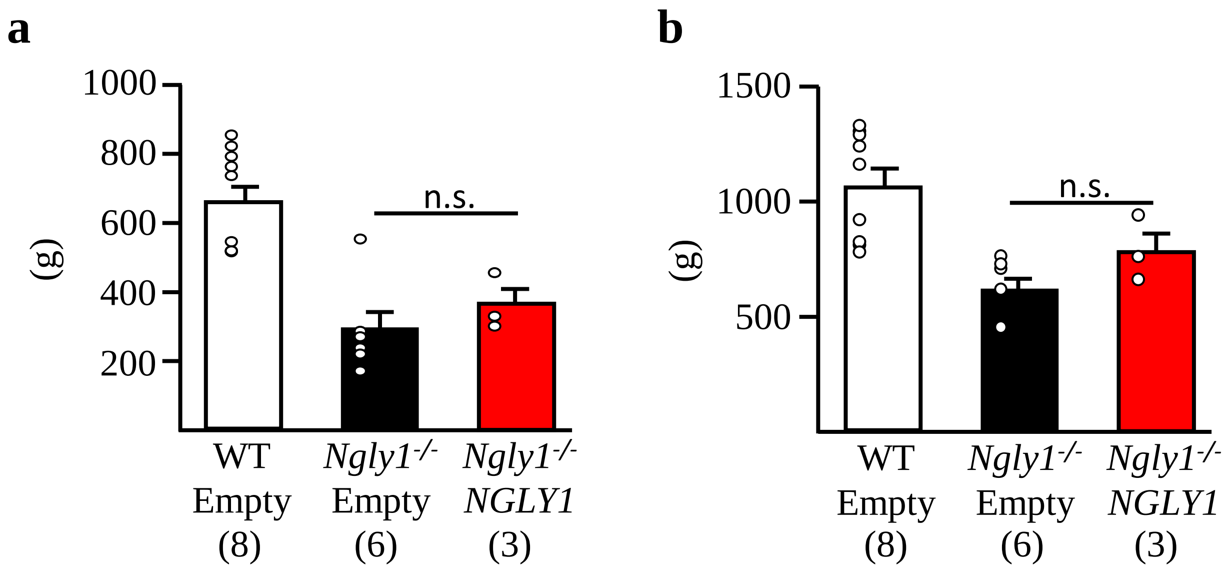

Additional file Fig. 4

Grip-strength tests for assessment of 2paws (**a**) or 4paws (**b**) muscle force of 11-week-old AAV-injected rats. Values represent means ± S.E.M. The number of rats examined is shown in parentheses. n.s. means no significance.

Additional file Fig. 5

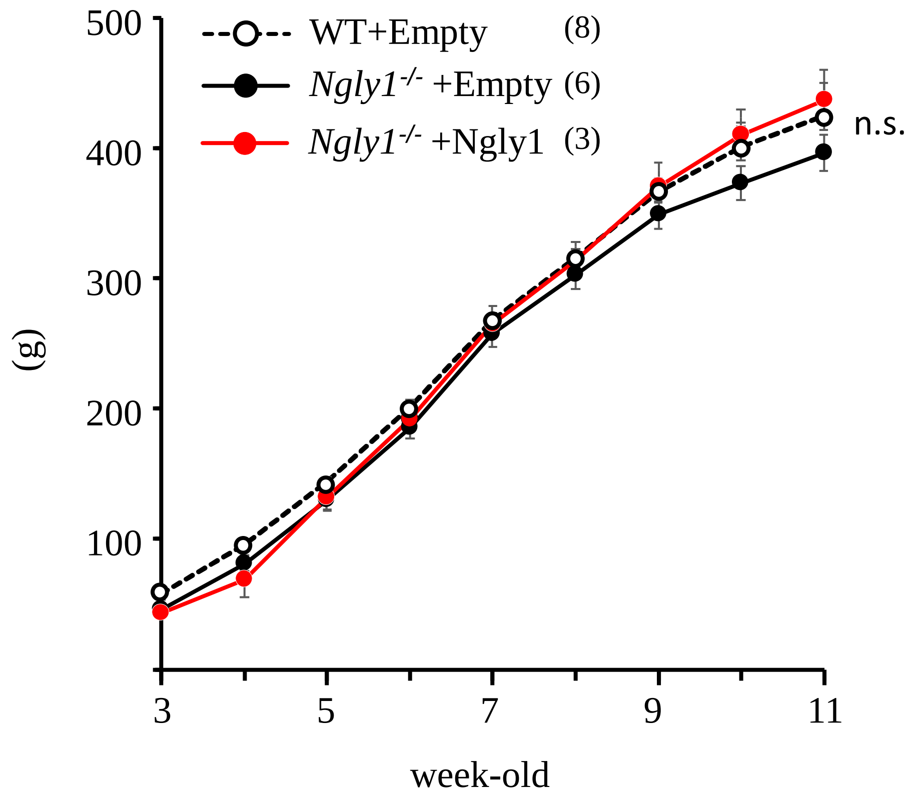

Additional file Fig. 5

Body weight of WT and *Ngly1−/−* rats. Rats were weighed weekly after AAV administration. The number of rats examined is shown in parentheses. n.s. means no significance.

Additional file Fig. 6

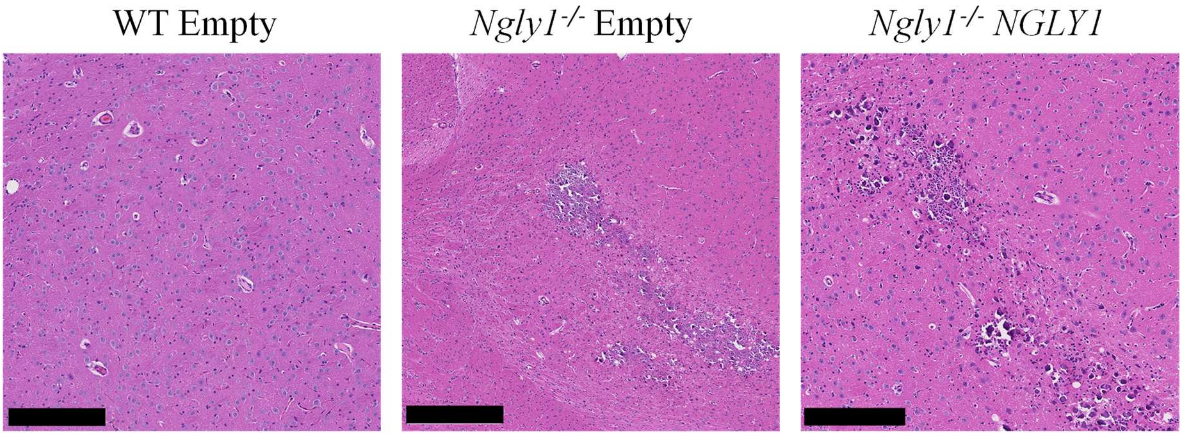

Additional file Fig. 6

Neuronal degeneration in the thalamus of *Ngly1*−/− rats was not recovered in AAV9-hNGLY1 injected *Ngly1*−/− rats. H&E-stained sections of the thalamus from the WT and *Ngly1*−/− rats 8 weeks after AAV administration. Scale bar 250 μm.

Additional file Fig. 7

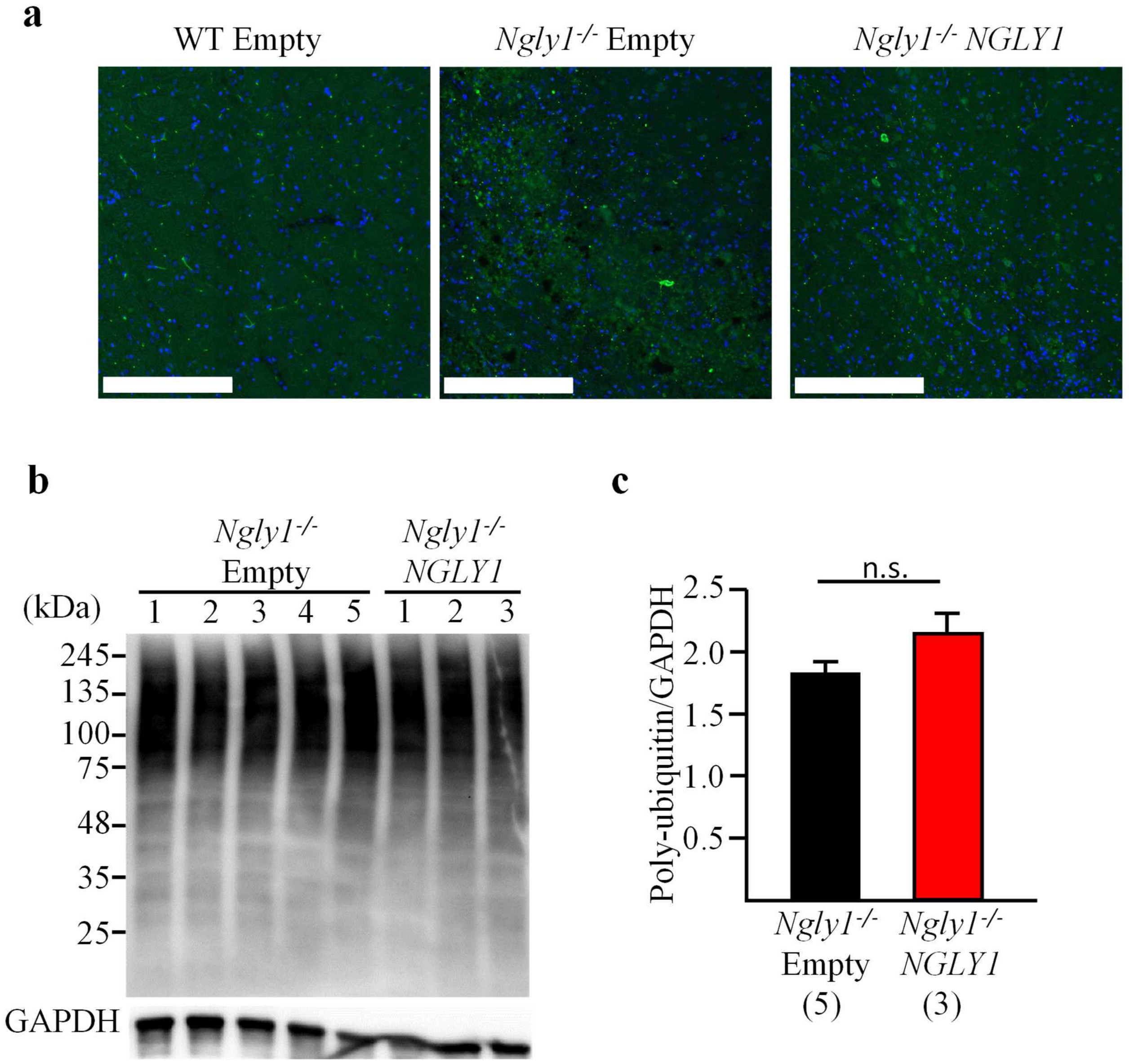

Additional file Fig. 7

(a) Immunohistochemistry for ubiquitin in the thalamus of virus-injected WT and Ngly1−/− rats at 11 weeks of age. Scale bar, 250 μm. Nuclei were stained with DAPI. **(b)** Accumulation of polyubiquitinated proteins in spinal cords of Ngly1−/− and WT rats. The total protein extracts were separated from the spinal cords of the rats and analyzed by immunoblotting using anti-polyubiquitinated antibodies (top) and anti-GAPDH antibodies (bottom; loading control). **(c)** Semi-quantitative analyses by densitometry were carried out. Values represent mean ± SEM (*Ngly1*−/− rats + empty; n = 5, *Ngly1*−/− rats + hNGLY1; n = 3). n.s. = no significance.
